# Analysis of Polycyclic Aromatic Hydrocarbons in Ambient Aerosols by Using One-Dimensional and Comprehensive Two-Dimensional Gas Chromatography Combined with Mass Spectrometric Method: A Comparative Study

**DOI:** 10.1155/2018/8341630

**Published:** 2018-04-01

**Authors:** Yun Gyong Ahn, So Hyeon Jeon, Hyung Bae Lim, Na Rae Choi, Geum-Sook Hwang, Yong Pyo Kim, Ji Yi Lee

**Affiliations:** ^1^Western Seoul Center, Korea Basic Science Institute, Seoul 03759, Republic of Korea; ^2^Air Quality Research Division, National Institute of Environmental Research, Incheon 22689, Republic of Korea; ^3^Department of Environmental Science and Engineering, Ewha Womans University, Seoul 03759, Republic of Korea; ^4^Department of Chemical Engineering and Material Science, Ewha Womans University, Seoul 03760, Republic of Korea

## Abstract

Advanced separation technology paired with mass spectrometry is an ideal method for the analysis of atmospheric samples having complex chemical compositions. Due to the huge variety of both natural and anthropogenic sources of organic compounds, simultaneous quantification and identification of organic compounds in aerosol samples represents a demanding analytical challenge. In this regard, comprehensive two-dimensional gas chromatography with time-of-flight mass spectrometry (GC×GC-TOFMS) has become an effective analytical method. However, verification and validation approaches to quantify these analytes have not been critically evaluated. We compared the performance of gas chromatography with quadrupole mass spectrometry (GC-qMS) and GC×GC-TOFMS for quantitative analysis of eighteen target polycyclic aromatic hydrocarbons (PAHs). The quantitative obtained results such as limits of detection (LODs), limits of quantification (LOQs), and recoveries of target PAHs were approximately equivalent based on both analytical methods. Furthermore, a larger number of analytes were consistently identified from the aerosol samples by GC×GC-TOFMS compared to GC-qMS. Our findings suggest that GC×GC-TOFMS would be widely applicable to the atmospheric and related sciences with simultaneous target and nontarget analysis in a single run.

## 1. Introduction

Human health research associated with polycyclic aromatic hydrocarbons (PAHs) has raised concerns because certain PAHs are classified as probable human carcinogens [1–4] and have shown tumorigenic activity and endocrine disrupting activity in mammals [5]. The US EPA has included 16 of them in the list of priority pollutants and has established a maximum contaminant level of 0.2 *μ*g/L for benzo[a]pyrene in drinking water [6]. In the European Union (EU), eight PAHs have been identified as priority hazardous substances in the field of water policy [7]. The EPA priority 16 PAHs and two additional PAHs are now being monitored by European agencies, and they have sought to quantify the individual concentrations of benzo[e]pyrene and perylene in environmental samples [6]. PAHs are found in ambient air in the gas phase and as sorbents to aerosols [8]. Thus, air monitoring of PAHs to quantify inhalation exposure and to identify other organic compounds is important for insight into photochemical reactions. The quantification and identification of organic compounds in air samples is an important feature of atmospheric chemistry and represents some demanding analytical challenges [9].

For these reasons, a key issue in current analytical methods is the ability to measure a large number of compounds with quantitative analysis for target analytes. Comprehensive two-dimensional gas chromatography (GC×GC) coupled with mass spectrometry (MS) can screen for nontarget compounds with fast identification of the compounds in an entire sample [10]. Therefore, previous studies applied GC×GC-MS for the identification of numerous compounds present in air samples [11–13]. However, there are limitations on the validation of simultaneous quantification and identification of analytes in air samples. Correspondingly, a validation of simultaneous identification and quantification of PAHs and other compounds in air samples by GC×GC–MS is required. A TOF mass spectrometer was used to acquire sufficient data from a comprehensive two-dimensional chromatographic technique that generated multiple narrow peaks from the short secondary column [14, 15]. Generally, GC coupled with quadrupole MS (GC-qMS) in the selected ion monitoring (SIM) mode has been used for quantitative analysis of PAHs in air samples because of its selective detection for specific target compounds [16, 17]. However, a GC×GC-TOFMS validated method suitable for the quantification of target PAHs in an aerosol sample compared with GC-qMS in the SIM mode has not yet been reported. The aim of this study was to evaluate the effectiveness of GC×GC-TOFMS in the quantitative analysis of target PAHs as well as the fast identification of multiple compounds for aerosol samples. The validity of the quantitative results obtained by both GC×GC-TOFMS and GC-qMS in the SIM mode was demonstrated by several method performance parameters such as linearity, accuracy, and repeatability.

## 2. Experimental

### 2.1. Air Sampling

The total suspended particle (TSP) samples were collected at Asan Engineering Building, Ewha Womans University, Seoul, South Korea (37.56°N, 126.94°E, 20 m above ground level), with a PUF sampler (Tisch, TE-1000) on a quartz fiber filter (Quartz fiber filter, QFF, Ø10.16 cm, Whatman, UK). The sampling site is located in the mixed resident area, commercial area, forest area, and nearby roadside. A total of 67 filter samples were obtained during summer (August 12–30, 2013) and winter (January 27–February 16, 2014) and day (9 a.m.∼6 p.m.) and night (8 p.m.∼6 a.m.). Prior to sampling, the quartz fiber filters were baked for 8 h in an electric oven at 550°C to remove possible organic contaminants. The sampled filters were wrapped in aluminum foils and stored in a freezer at −20°C until analysis.

### 2.2. Chemicals

All organic solvents were of GC grade and purchased from Burdick and Jackson (Phillipsburg, NJ, USA). Standard solutions of target PAHs (Table 1 for their full chemical names and information) except Per and BeP for quantitative analysis were purchased as a mixture at a concentration of 2000 *µ*g/mL in dichloromethane from Supelco (Bellefonte, PA, USA). Per and BeP standards (>99%) were purchased from Aldrich (St. Louis, MI, USA), and a standard mixture of eighteen PAHs was prepared at a concentration of 1000 *µ*g/mL. Deuterium-labeled internal standards of seven PAHs were purchased from Aldrich (St. Louis, MI, USA) and Chiron (Trondheim, Norway) and used for the spiking test as listed in Table 1. Working standard solutions (0.01∼10 *µ*g/mL) were prepared and then stored at −20°C prior to use.

### 2.3. Preparation of Samples

Air sampling filters were extracted with a mixture of dichloromethane and methanol (3 : 1, v/v) two times using an accelerated solvent extractor (ASE) (Dionex ASE-200) at 40°C and 1700 psi for 5 min. Prior to the extraction, seven deuterated internal standards (Nap-d8, Ace-d10, Phen-d10, Fla-d10, Chr-d12, Per-d12, and BghiPer-d12) were spiked in the filters to compensate for matrix effects during the extraction procedure. Extracts were blown down to 1 mL using a nitrogen evaporator (TurboVap II, Caliper Life Sciences). GC×GC-TOFMS analysis was carried out using an Agilent GC (Wilmington, Delaware, USA)-Quad-jet thermal modulation Pegasus 4D TOFMS (LECO, St. Joseph, MI, USA). The sample was injected in the splitless mode at 300°C. The GC×GC columns were as follows: DB-5MS (30 m × 0.25 mm ID, film thickness of 0.25 *μ*m) and 1.17 m DB-17MS (0.18 mm OD, 0.18 *μ*m film). The operating conditions of GC-MS and GC×GC-TOFMS are summarized in Table 2.

## 3. Results and Discussion

### 3.1. GC-qMS and GC×GC-TOFMS for Characterization of Aerosol Samples

**Figure 1:**
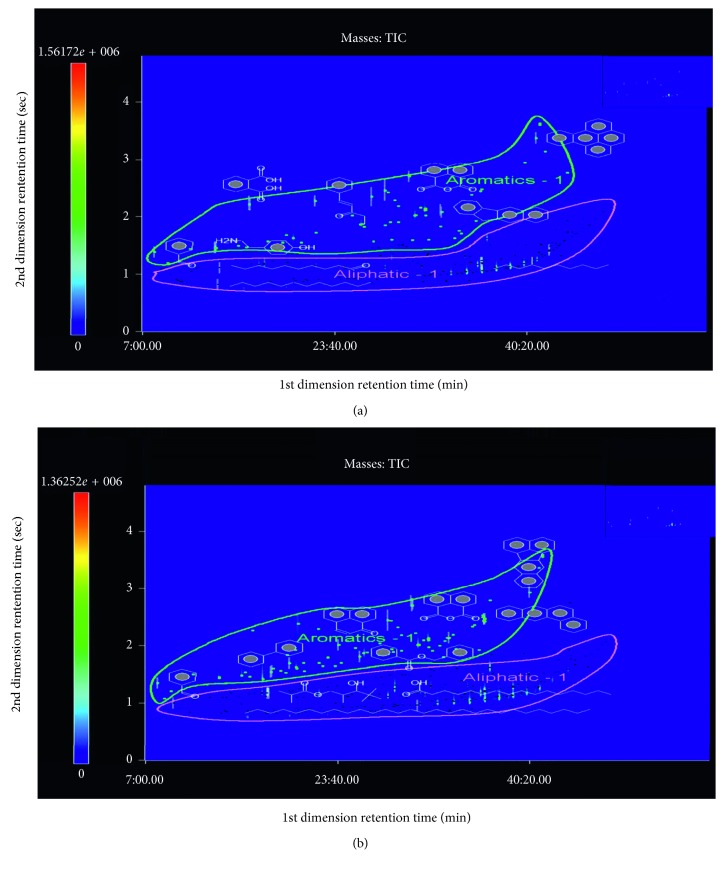
GC×GC-TOFMS plots of aerosol samples collected during day (a) and night (b) of winter in Seoul, Korea. A total of 251 and 297 peaks were identified in aerosol samples collected during day (a) and night (b), respectively. Aromatic and aliphatic classes were drawn to divide two regions for ease of viewing.

**Figure 2:**
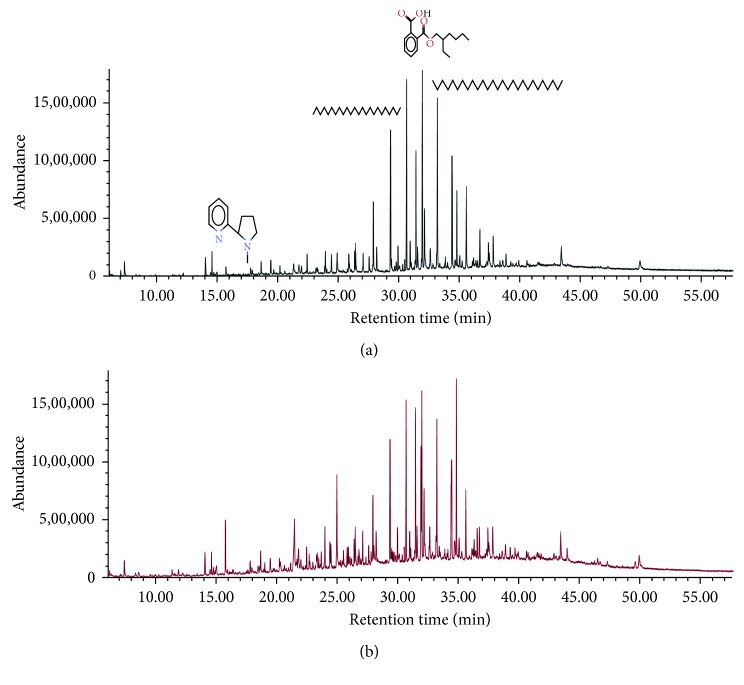
Total ion chromatograms of aerosol samples collected in day (a) and night (b) of winter in Seoul, Korea, obtained by GC-qMS. A total of 35 and 64 peaks were identified in aerosol samples collected during day (a) and night (b), respectively. The analytes were separated based on their boiling points.

In most studies, separation and quantification of PAHs in aerosol samples have been analyzed using a conventional GC-qMS [18]. Flame ionization detection (FID) has also been widely used for quantification as it features a higher response to PAHs which contain only carbon and hydrogen, while oxygenates and other species that contain heteroatoms tend to have a lower response factor [19]. However, this nonspecific detector may not distinguish inferences, which include a large fraction of aliphatic and aromatic compounds in aerosol samples from alkylated PAH homologues. The coupling of GC with MS is increasingly becoming the analytical tool of choice in this regard because of its superior selectivity and sensitivity. Among the most common analyzers including TOF [20], ion trap, and qMS [21, 22], qMS is the most widely adopted technique for routine analysis of PAHs [23]. GC-qMS data acquisition takes advantages of both a full mass scan range (scan mode) and specific ion masses for target analytes (SIM mode). The sensitivity in the SIM mode is higher than that in the scan mode of GC-qMS due to the increased dwell time on each monitored ion for trace analysis in some matrices such as in atmospheric aerosols [24, 25]. GC-TOFMS has a much faster spectral acquisition rate than GC-qMS does, which is up to 500 full mass scans per second [26]. Consequently, this system is able to widen the application of GC×GC techniques providing very narrow chromatographic peaks, typically 50∼600 ms at the baseline with sufficient density of data points per chromatographic peak [27]. Environmental samples are generally complex, often with more than hundreds of compounds containing structural isomers and homologues spread over a wide range of concentration and volatility. Accordingly, multidimensional separation is an advanced technique offering the possibility of greatly enhanced selectivity using different separation mechanisms for the analysis of complex environmental samples [28–30]. In this study, a set of columns DB-5×DB-17 ms was applied to increase the resolution and peak capacity. The fast scanning Pegasus 4D TOFMS system was combined to allow efficient processing of data acquisition, handling, peak detection, and deconvolution. In the one-dimensional column, a 30 m-long DB-5 ms (5% diphenyl/95% dimethyl polysiloxane) stationary phase was used to separate analytes based on volatility and combined with a 1.17 m-long DB-17 ms column (50% diphenyl/50% dimethyl polysiloxane) allowing relative polarity-based separation.  Figure 1 shows GC×GC-TOFMS chromatograms of aerosol samples collected at day and night during winter in Seoul, South Korea. To compare the identification ability of GC×GC-TOFMS with GC-qMS, analysis with GC-qMS in the scan mode was performed. A comparison of the one-dimensional chromatograms of the same samples obtained by GC-qMS is shown in Figure 2. 2D chromatograms enable the visual classification of chemically related compounds into groups. It was rare to see that the early-eluting analytes have an extreme volatility in the chromatogram, as shown in Figure 2. Because of the large losses of these analytes during sample extraction and concentration, particle-associated semivolatile analytes were mainly detected and classified according to their aromatic and aliphatic hydrocarbon groups.

**Figure 3:**
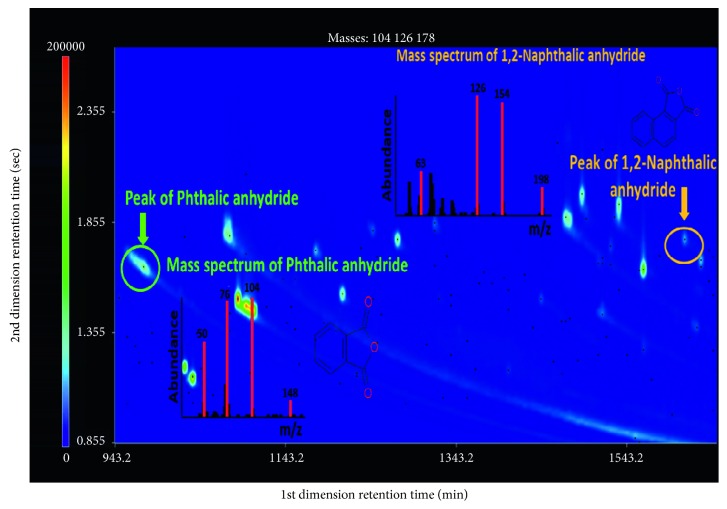
GC×GC chromatograms and mass spectrums of phthalic anhydride (marked as green) and 1,2-naphthalic anhydride (marked as yellow) in the aerosol sample. GC×GC chromatograms of phthalic anhydride and 1,2-naphthalic anhydride were certified by molecular ions of *m*/*z* 148 and 198, respectively.

Meanwhile, analytes from the GC-qMS chromatogram were separated based on their vapor pressures or boiling points. The GC×GC technique is rather well suited for group separations, and classifying compounds into chemical-related groups could be useful for source identification of atmospheric aerosols by means of the large amount of chemical data handling. The combined use with TOFMS provides rapid and reliable identification of analytes using their deconvoluted pure mass spectra. The major limitation of qMS is its limited scan rate; therefore, quantification and identification is seriously compromised because of the mass spectral skew due to the variations in ion abundances at different regions of a chromatographic peak [31, 32]. The numbers of identified chromatographic peaks analyzed by GC-qMS using the same signal threshold setting from the aerosol samples collected at day and night were 35 and 64, respectively. In the case of results obtained by GC×GC-TOFMS, 251 and 297 peaks from the day- and night-time aerosol samples were, respectively, assigned by individual spectral deconvolution. As a result, phthalic anhydride and 1,2-naphthalic anhydride as the markers of secondary formation for gas-phase PAH reactions were identified in the aerosol sample, as shown in Figure 3. Since the products formed through photochemical reactions are often more toxic than their parent PAHs in atmosphere [17], significant efforts have been expended to identify the photochemical products with PAHs in the fields of atmospheric or environmental sciences. In the case of results obtained using GC-qMS, phthalic anhydride and 1,2-naphthalic anhydride were not detected in the same sample. Limitations of one-dimensional separation have been reported for these photochemical products and complex mixtures of the aerosol sample because of their diverse polarities in a single run [33, 34]. Contrastively, two anhydrides associated with secondary organic aerosol formation were clearly separated and detected by GC×GC-TOFMS. Therefore, it showed advantages for nontarget screening to identify molecular markers or chemical patterns more representative of the aerosol state observed in ambient air.

### 3.2. Validation of GC-qMS and GC×GC-TOFMS for Quantification of PAHs

GC-qMS and GC×GC-TOFMS were tested individually in order to evaluate their analytical performances. The calibration linearity (regression coefficient, *R*^2^) and relative response factor (RRF) are presented in Table 3. The RRF is the ratio between a signal produced by an individual native analyte and the corresponding isotopically labeled analogue of the analyte (as an internal standard). For calculating RRF, 2 ng of each target PAH and each corresponding deuterated internal standard was spiked, and the relative sensitivity in both the methods was compared. Despite the high-speed scanning performance of GC×GC-TOFMS, the RRFs obtained by this method were approximately equivalent to those obtained by GC-qMS. RRF expresses the sensitivity of a detector for a given substance relative to a standard substance [35, 36]. Thus, it indicated that the sensitivity of GC×GC-TOFMS relative to target PAHs is comparable in quantitative analysis. Calibration curves were generated using the peak area for the 18 PAHs at seven concentrations ranging from 0.01 to 10 *μ*g/mL. The linearity was assessed by calculating the regression equation and the correlation coefficient by the least squares method, as shown in Table 3. The *R*^2^ values were greater than 0.999 for GC-qMS and 0.99 for GC×GC-TOFMS. Although data processing for quantification by GC×GC-TOFMS was derived from the combined peak areas for the slices of modulated peaks in contrast to production of the single measured peak by GC-qMS, the results meet the criteria for acceptable linearity within this calibration range. Naturally, the development of quantitative GC×GC studies based on the quantitative results associated with sophisticated implementation for modulated peaks has been delayed compared with qualitative reports. Recently, the approach to quantifying multiple analytes at once with comprehensive two-dimensional GC has been extensively studied in accordance with the improvement of data processing for the integration of modulated peaks [37, 38]. In this study, the modulated peaks of each PAH was automatically combined and integrated by the ChromaTOF software based on a similarity of spectra within an allowable time difference between the second dimension peaks in the neighboring slices of the chromatogram. Recovery test was performed by spiking known amounts of the 18 PAH compounds in a prebaked clean filter at a final concentration of 2 *μ*g/mL and analyses of each through all the experiment procedures were compared using the two different methods. Six duplicate tests were performed, and the results of the recovery are shown in Table 4. The average recoveries were in the range of 90.3 to 158% with relative standard deviations (RSDs) ranging from 3.9 to 28% for GC-qMS, while the recoveries were from 86.3 to 135% for GC×GC-TOFMS, with RSDs ranging from 5.7 to 45%. Most of the targeted PAH compounds were afforded acceptable recoveries, excluding F and Nap by using the two analytical methods due to the high volatility of these compounds. Compared with the reproducibility as expressed in %RSDs, the values obtained by GC-qMS were slightly lower than those obtained by GC×GC-TOFMS; however, the %RSD values of the targeted PAHs excluding F and Nap were acceptable (<20% RSD). These observations may vary for the versatile GC×GC technique, since the reproducibility of the modulation phase is dependent on the type of modulator, the stability of the stationary phases, and the chemistry of the analyte, regarding interaction with the stationary phase as presented in several prior studies [39, 40]. The LOD and LOQ were determined based on the standard deviation (SD) of the intersection of the analytical curve (*s*) and the slope of the curve (*S*) as LOD = 3.3 × (*s*/*S*) and LOQ = 10 × (*s*/*S*). The LOD and LOQ for each PAH compound obtained from both the methods are shown in Table 4. The LOD and LOQ values of the 18 PAH compounds obtained by GC-qMS were similar to the results of previous studies [10, 41, 42]. Thus, the suitability of GC×GC-TOFMS for quantification of PAHs was proven by comparing the results with those obtained using GC-qMS.

## 4. Conclusion

A fast scanning GC×GC-TOFMS was compared to a GC-qMS for the determination of PAHs in aerosol samples. For separation, identification, and characterization, GC×GC-TOFMS was advantageous over GC-qMS owing to the increased peak capacity, and its results showed enhanced detectability and structured chromatograms for nontarget analysis. The qualitative mass separation by TOFMS combined with an automated peak-finding capability provided the resolution of complex mixed mass spectra, resulting from overlapping chromatographic peaks and spectral deconvolution of individual mass spectra for unknown analytes. Furthermore, the obtained quantitative results such as LODs, LOQs, and recoveries of the 18 target PAHs were approximately equivalent for both the analytical methods. Thus, GC×GC-TOFMS had advantages for the simultaneous quantification and qualification of PAHs and other organic compounds in a single run. Because of its high degree of separation and capability of spectral deconvolution of overlapping peaks in highly complex samples, comprehensive GC×GC-TOFMS may become a useful platform in many other fields of research.

## Figures and Tables

**Table 1:** Information of target PAHs in the study.

Compound	Abbreviation	CAS number	Molecular formula	MW	Quantitative ion	Qualifier ion	Retention time		
							GC-qMS	GC×GC-TOFMS	
							(min)	*t*_*r*1_ (min)	*t*_*r*2_ (s)
Naphthalene-d_8_^a^	Nap-d_8_	1146-65-2	C_10_D_8_	136.2	136	137	12.25	13.40	1.34
Naphthalene	Nap	91-20-3	C_10_H_8_	128.2	128	129	12.34	13.47	1.35
Acenaphthylene	Acy	208-96-8	C_12_H_8_	152.2	152	153	19.08	19.56	1.55
Acenaphthene-d_10_^a^	Ace-d_10_	15067-26-2	C_12_D_10_	164.2	162	164	19.47	20.12	1.52
Acenaphthene	Ace	83-32-9	C_12_H_10_	154.2	153	154	19.61	20.28	1.52
Fluorene	F	86-73-7	C_13_H_10_	166.2	166	165	21.61	22.28	1.53
Phenanthrene-d_10_^a^	Phen-d_10_	1518-22-2	C_14_D_10_	188.2	188	189	25.91	25.96	1.68
Phenanthrene	Phen	85-01-8	C_14_H_10_	178.2	178	179	26.01	26.04	1.71
Anthracene	Ant	120-12-7	C_14_H_10_	178.2	178	179	26.14	26.20	1.68
Fluoranthene-d_10_^a^	Fla-d_10_	93951-69-0	C_16_D_10_	212.2	212	213	30.99	30.68	1.84
Fluoranthene	Fla	206-44-0	C_16_H_10_	202.2	202	203	31.08	30.68	1.87
Pyrene	Pyr	129-00-0	C_16_H_10_	202.2	202	203	32.25	31.56	1.98
Benz[a]anthracene	BaA	56-55-3	C_18_H_12_	228.2	228	226	37.20	36.28	2.21
Chrysene-d_12_^a^	Chr-d_12_	1719-03-5	C_18_D_12_	240.3	240	236	37.42	36.28	2.27
Chrysene	Chr	218-01-9	C_18_H_12_	228.3	228	226	37.54	36.44	2.26
Benzo[b]fluoranthene	BbF	205-99-2	C_20_H_12_	252.3	252	253	41.55	40.12	2.76
Benzo[k]fluoranthene	BkF	207-08-9	C_20_H_12_	252.3	252	253	41.65	40.28	2.74
Benzo[e]pyrene	BeP	192-97-2	C_20_H_12_	252.3	252	253	42.90	41.08	3.16
Benzo[a]pyrene	BaP	50-32-8	C_20_H_12_	252.3	252	253	43.09	41.24	3.23
Perylene-d_12_^a^	Per-d_12_	1520-96-3	C_20_D_12_	264.3	264	260	43.46	41.40	3.36
Perylene	Per	198-55-0	C_20_H_12_	252.3	252	253	43.57	41.48	3.47
Indeno[1,2,3-cd]pyrene	IP	193-39-5	C_22_H_12_	276.3	276	277	48.09	45.48	0.71
Dibenz[a,h]anthracene	DBahAnt	53-70-3	C_22_H_14_	278.3	278	279	48.12	45.64	0.82
Benzo[ghi]perylene-d_12_^a^	BghiPer-d_12_	93951-66-7	C_22_D_12_	288.3	288	284	49.92	46.52	1.54
Benzo[ghi]perylene	BghiPer	191-24-2	C_22_H_12_	276.3	276	277	50.13	46.68	1.78

^a^Internal standard.

**Table 2:** GC-qMS and GC×GC-TOFMS operating conditions.

Parameters	GC-qMS	GC×GC-TOFMS
*Injector settings*		
Injection volume	1 *μ*L	1 *μ*L
Inlet mode	Splitless	Splitless
Carrier gas	He (99.999%)	He (99.999%)
Carrier gas flow	1.0 mL·min^−1^	1.3 mL·min^−1^
Inlet temperature	280°C	300°C
*GC oven temperature*		
Initial temperature	1 min at 60°C	1 min at 60°C
First rate	6°C/min to 310°C	6°C/min to 300°C
Isothermal pause	15 min at 310°C	15 min at 300°C
2nd oven temperature offset	—	5°C, relative to the 2nd oven temperature
*Modulator*		
Modulator temperature offset	—	15°C, relative to the 2nd oven temperature
Modulator period	—	4.00 s
Hot pulse time	—	1.00 s
Cool time between stages	—	1.40 s
*MS*		
Mass range	40∼550	40∼550
Electron energy	70 eV	70 eV
Ion source temperature	230°C	230°C

**Table 3:** Relative response factors (RRFs) and calibrations of 18 PAHs obtained by the compared methods.

Compound	GC-qMS				GC×GC-TOFMS			
	RRF^a^	Slope	Intercept	*R* ^2^	RRF	Slope	Intercept	*R* ^2^
Nap	1.04	0.515	−0.003	0.9999	1.69	0.560	0.049	0.9971
Acy	1.57	0.808	−0.004	1.0000	1.95	1.026	−0.012	0.9999
Ace	1.03	0.439	0.009	0.9999	1.16	0.553	−0.006	0.9994
F	1.29	0.667	−0.006	1.0000	1.11	0.609	−0.021	0.9997
Phe	1.17	0.572	−0.004	0.9998	1.47	0.763	−0.039	0.9979
Ant	0.98	0.547	−0.017	0.9992	0.93	0.431	−0.010	0.9982
Fla	1.30	0.678	−0.001	1.0000	1.43	0.802	−0.021	0.9995
Pyr	1.31	0.686	−0.004	0.9999	1.62	0.910	−0.055	0.9972
BaA	0.98	0.575	−0.019	0.9997	1.42	0.574	−0.004	0.9998
Chr	1.06	0.563	−0.003	1.0000	1.26	0.651	−0.005	0.9998
BbF	0.99	0.535	−0.008	0.9999	1.69	0.848	−0.028	0.9993
BkF	1.11	0.576	−0.011	0.9998	0.88	0.327	−0.012	0.9977
BeP	0.91	0.455	−0.007	0.9996	0.90	0.530	−0.014	0.9997
BaP	0.88	0.505	−0.014	0.9997	0.83	0.477	−0.030	0.9985
Per	0.89	0.475	−0.008	0.9998	1.11	0.521	-0.023	0.9979
IP	1.37	0.717	−0.023	0.9995	1.25	0.660	-0.062	0.9922
DBahAnt	1.24	0.629	−0.019	0.9996	1.16	0.490	-0.074	0.9898
BghiPer	1.24	0.594	−0.010	1.000	1.53	0.710	-0.036	0.9991

^a^RRF expresses the sensitivity of a detector for a given analyte relative to its corresponding deuterated internal standards; RRF=(*A*_*x*_*C*_is_)/(*A*_is_*C*_*x*_), where *A*_*x*_ is the peak area of a quantifying ion for a given analyte being measured; *A*_is_ is the peak area of a quantifying ion for its corresponding internal standard; *C*_*x*_ is the concentration of a given analyte; and *C*_is_ is the concentration of its corresponding internal standard.

**Table 4:** Limits of detection and quantification and recoveries of 18 PAHs obtained by the compared methods.

Compound	LOD^a^ (ng)		LOQ^b^ (ng)		Recovery ± RSD (%)	
	GC-qMS	GC×GC-TOFMS	GC-qMS	GC×GC-TOFMS	GC-qMS	GC×GC-TOFMS
Nap	0.07	0.40	0.21	1.19	94.4 ± 4.2	135 ± 45
Acy	0.17	0.07	0.51	0.22	119 ± 12	116 ± 15
Ace	0.05	0.17	0.16	0.52	105 ± 5.3	105 ± 7.8
F	0.04	0.15	0.13	0.44	158 ± 28	130 ± 29
Phe	0.10	0.34	0.31	1.03	94.5 ± 5.3	86.3 ± 16
Ant	0.19	0.31	0.58	0.92	90.4 ± 4.6	95.1 ± 20
Fla	0.05	0.14	0.16	0.41	90.3 ± 3.9	105 ± 13
Pyr	0.08	0.36	0.25	1.09	97.4 ± 5.3	97.2 ± 13
BaA	0.12	0.09	0.37	0.27	93.4 ± 4.9	86.9 ± 8.2
Chr	0.04	0.08	0.13	0.24	95.8 ± 5.8	101 ± 16
BbF	0.05	0.18	0.15	0.53	96.1 ± 5.7	92.3 ± 10
BkF	0.09	0.35	0.28	1.05	94.2 ± 6.5	105 ± 12
BeP	0.13	0.13	0.40	0.38	92.6 ± 5.8	92.7 ± 5.7
BaP	0.12	0.24	0.37	0.72	93.6 ± 5.3	104 ± 9.0
Per	0.11	0.34	0.32	1.02	93.0 ± 5.5	92.5 ± 8.6
IP	0.15	0.65	0.16	1.94	95.0 ± 5.4	93.9 ± 8.5
DBahAnt	0.13	1.05	0.40	3.14	94.9 ± 5.5	95.8 ± 5.7
BghiPer	0.09	0.22	0.27	0.66	94.6 ± 6.0	87.0 ± 8.5

^a^LOD, smallest amount of analyte that is statistically different from the blank; ^b^LOQ, smallest amount of analyte that can be measured with reasonable accuracy.
